# Functional olfactory evolution in *Drosophila suzukii* and the subgenus *Sophophora*

**DOI:** 10.1016/j.isci.2022.104212

**Published:** 2022-04-06

**Authors:** Ian W. Keesey, Jin Zhang, Ana Depetris-Chauvin, George F. Obiero, Abhishek Gupta, Nitin Gupta, Heiko Vogel, Markus Knaden, Bill S. Hansson

**Affiliations:** 1 Max Planck Institute for Chemical Ecology, Department of Evolutionary Neuroethology, Hans-Knöll-Straße 8, D-07745 Jena, Germany; 2 Department of Biochemistry and Biotechnology, Technical University of Kenya, Haille-Sellasie Avenue, Workshop Rd, 0200 Nairobi, Kenya; 3 Department of Biological Sciences and Bioengineering, Indian Institute of Technology Kanpur, Kanpur, Uttar Pradesh 208016, India; 4 Department of Chemistry, Indian Institute of Technology Kanpur, Kanpur, Uttar Pradesh 208016, India; 5 Max Planck Institute for Chemical Ecology, Department of Entomology, Hans-Knöll-Straße 8, D-07745 Jena, Germany

**Keywords:** agricultural science, Entomology, evolutionary biology, sensory neuroscience

## Abstract

Comparative analyses of multiple genomes are used extensively to examine the gains and losses of chemosensory receptors across the genus *Drosophila*. However, few studies have delved into functional olfactory characteristics. Here we assess olfactory function across 20 species, and identify and describe several similar elements of evolution. We document (a) minor changes in functional ligands based on amino acid substitutions, (b) major changes in olfactory function or perhaps entire receptor replacements, and (c) that only a few receptors are subject to repeated changes, whereas 32 out of 37 OSNs are largely functionally conserved. In addition, we generate a robust model for identifying olfactory function using genomic data and comprehensive ligand-receptor combinations, which includes the prediction of binding pockets. Moreover, this study highlights that functional olfactory evolution does not affect all chemosensory receptors equally, and that ecological, evolutionary, and developmental forces repeatedly affect only a small subset of available receptor proteins.

## Highlights

• We complete an overview of *Drosophila suzukii* olfaction across both the antenna and palps
• We examine the evolution of chemosensation across 20 species within *Sophophora*
• We identify sensillum and potential receptor types shifting in their function
• Evolution often alters both OR abundance and OR sequence concurrently

## Graphical abstract

**Figure:**
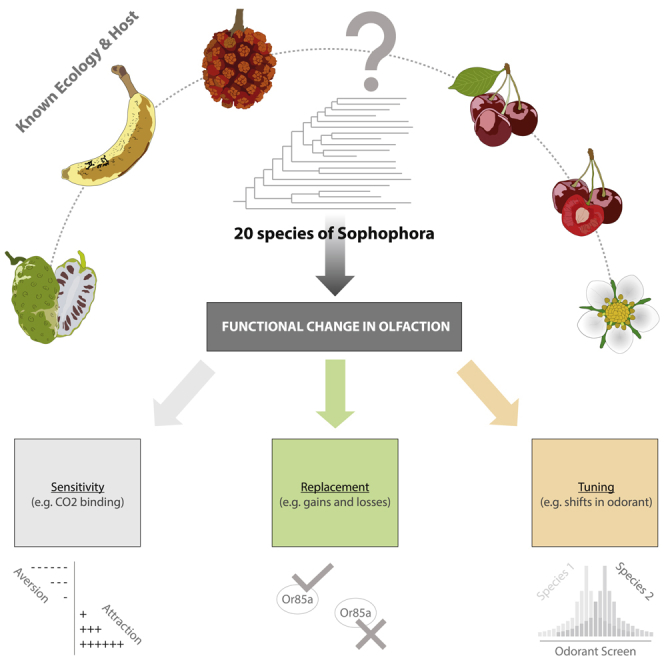

## Introduction

One of the advantages of working within the *Drosophila* genus is the wide array of evolutionary specializations that these species display, especially in regard to host preference, but also habitat choice, morphology, and mate selection (Keesey et al., 2019a; Markow, 2015). As an additional benefit, the genus affords a vast amount of genomic data generated and accumulated since the early 21st century (Adams et al., 2000; Clark et al., 2007; Khallaf et al., 2021). The last ten years of research have also brought attention toward another novelty, an agricultural pest species in the form of *D. suzukii*, which has now invaded North and South America, Europe as well as its native Asian origins (Asplen et al., 2015; Cini et al., 2012; Deprá et al., 2014; Lee et al., 2011; Walsh et al., 2011). Furthermore, this pest insect has prompted both integrated pest management efforts as well as evolutionary neuroethology research, where the distinct ecological niche of attacking fresh or ripening fruit has afforded a unique opportunity for comparison among the other model species within this well-studied genus of flies. Thus, whereas most species within the subgenus *Sophophora* usually only target rotten, softened, or fermented host resources, *D. suzukii* has been shown repeatedly to target fresh, hardened, and ripe fruits for feeding and oviposition (Karageorgi et al., 2017; Keesey et al., 2015, 2019b; Maderspacher and Stensmyr, 2017).

Previous research has sought to outline either the olfactory or gustatory systems of several species of *Drosophila*, either for evolutionary or ecological comparisons of the host plant, mate preference, or reproductive isolation, including *Drosophila melanogaster*, *Drosophila sechellia*, and *Drosophila simulans* (Auer et al., 2020; Fan et al., 2013; Hallem and Carlson, 2006; Seeholzer et al., 2018). However, only a few studies have conducted electrophysiological assessments of members outside of this subgroup (Crowley-Gall et al., 2016; de Bruyne et al., 2010), with most research examining non-*melanogaster* species only in regards to a single, specific olfactory sensory neuron (OSN) of interest. Moreover, usually only as a means to show the conserved nature of that functional neuronal type (Dekker et al., 2006, 2015; Ebrahim et al., 2015; Linz et al., 2013; Stensmyr et al., 2012). Some more distantly related *Drosophila* species have begun to be examined in more depth, such as *Drosophila mojavensis* (Crowley-Gall et al., 2016, 2019; Khallaf et al., 2020, 2021). This species is an excellent model for incipient speciation; however, this species is a member of an entirely different subgenus, and, as such, is quite far removed from the more robust data sets afforded by the *melanogaster* clade and the other members of *Sophophora* subgenus. These factors may also mean that these distantly related species (i.e., *D. mojavensis* and the *Drosophila* subgenus) are much more difficult to utilize to assess patterns and mechanisms of evolutionary selective pressure, at least in their direct comparison with the more established molecular model species within the *melanogaster* clade. Therefore, in contrast, the non-*melanogaster* members of the *Sophophora* subgenus, which itself includes substantial variation in host and habitat choice, perhaps represents a closer group of relatives to optimize the comparisons of evolutionary variation in chemosensation. Here again, *D. suzukii* and its related subgroups offer an ideal, genetically tractable model for unraveling the complexities of olfactory evolution, especially given their relative phylogenetic proximity to the principal scientific models within the *melanogaster* clade (Keesey et al., 2019a; Markow, 2015).

For many *Drosophila* species, there already exists a robust library of molecular resources, as nearly complete genomic data sets are publicly available; however, far less information is available describing the functional components of host ecology, such as olfactory, gustatory, auditory and visual preferences, or describing behavioral or habitat variations between these species. Thus, for most members across this incredibly well-studied genus, more is known about their genome than their ecology. In an effort to expand our knowledge about these species, here we provide a functional olfactory comparison encompassing 20 different species within the *Sophophora* subgenus, with particular focus on *D. suzukii* and its closest relatives, in order to examine the variables and olfactory attributes that are associated with the evolutionary emergence of this insect pest. In addition, we address how protein sequence coding of olfactory receptors correlates with the functional evolution of odorant binding and ligand selectivity, through comparison of chemosensory variation overlaid with tertiary protein structures from the available species.

Thus, in summary, the present study establishes the missing ingredients to begin understanding the trends and mechanisms of olfactory evolution, whereas also providing critical chemosensory evaluations and a foundation of knowledge across 20 species within this genus of insects. We show that only a few OSNs of the identified orthologues between species typically display elevated levels of sequence divergence as well as functional variation between species, including both large and small deviations in olfactory responses at the periphery. This study also proposes that the comparative approach, which examines olfactory function as well as amino acid or receptor sequence alterations and patterns of selection across a multitude of species, may provide further insight into the evolution of sensory systems. Moreover, the identified OSN locations within this study (and the potential ORs that they contain) appear to repeatedly be important for further examination across the subgenus *Sophophora*, and will be potential targets for future research concerning the mechanisms of chemosensory divergence and ecological adaptation.

## Results

### Complete screening of *D. suzukii* olfaction

**Figure 1:**
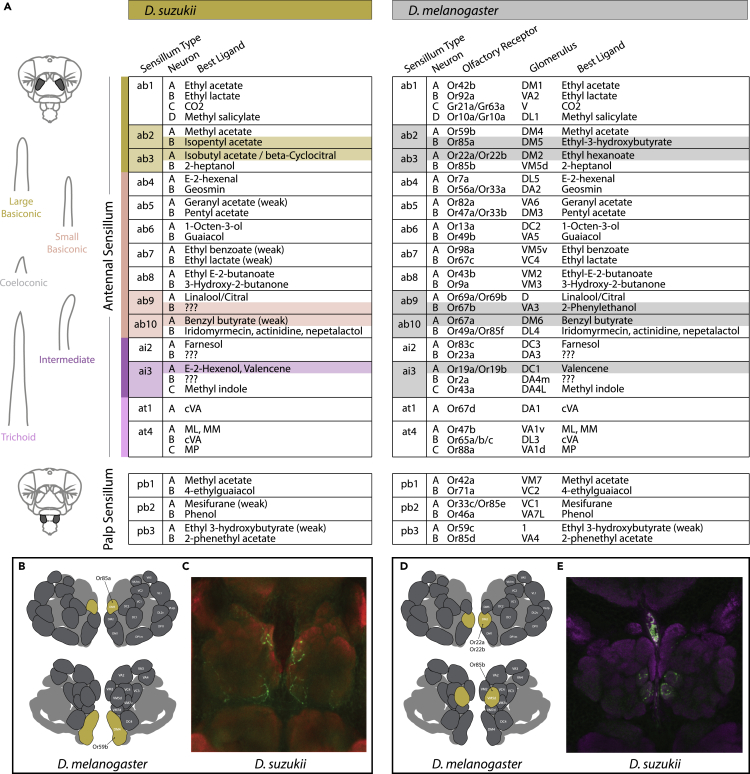
Complete olfactory sensory neuron screen of *D. suzukii* (A) Information for each olfactory channels and sensillum type found across antenna or palps. In total, 37 neurons were analyzed from each species using single sensillum recordings (SSR), where 32 out of 37 of the receptors retain the same ligand spectra, whereas only five OSNs (14%) displayed variation. Responses that were different are highlighted. “Best ligand” is the strongest excitatory ligand in the screen. (B–E) Immunostaining with neurobiotin (green) and nc82 (red or magenta) across the antennal lobes of *D. suzukii* adults that were backfilled from the ab2- or ab3-like sensillum types. Each sensillum type was identified with electrophysiological contact and odor response recordings before the application of the neurobiotin. (B) Spatial map of the antennal lobe (AL) atlas of *D. melanogaster* of neurons from ab2 sensillum. (C) Neuronal wiring in *D. suzukii* that stem from single-sensillum backfills of ab2-like sensillum, showing similar mapping. (D) Spatial map of the AL of *D. melanogaster* of neurons from ab3 sensillum. (E) Neuronal wiring in *D. suzukii* from single-sensillum backfills of ab3-like sensillum. This spatial congruence, combined with the morphological (e.g. large basiconic) and odor ligand data from the other neuron housed in either ab2 or ab3 sensillum (e.g. Or59b and Or85b), continues to provide strong, corroborating evidence that the wiring for *D. suzukii* and *D. melanogaster* is unchanged. This conservation of wiring within the antennal lobe is despite changes in odorant sensitivity and ligand spectra for the ab2B and ab3A OSNs between these two species.

In order to assess olfactory ligand spectra for all OSN types, we first revisited the classical model, *D. melanogaster*, where the responses of most OSNs have been previously established (Crowley-Gall et al., 2016, 2019; Dweck et al., 2016; Hallem and Carlson, 2006; Lin and Potter, 2015; Mathew et al., 2013; van der Goes van Naters and Carlson, 2007). Here we again mapped out the response profile from each known receptor across the adult fly antenna and palps using a panel of 80 odorants (Figure 1). We found that out of 37 unique olfactory sensory neurons (OSNs), only 3 are still currently without a strong ligand candidate (i.e., Or23a, Or2a, Or65a/b/c), although, in addition, several co-expressed receptors also have poorly defined response profiles (e.g., Or33a, which is found in OSN ab4B). Next, we shifted our focus toward the antenna and palps of *D. suzukii* using the same diverse panel of 80 odorants in order to examine any changes in functional ligand spectra between these two species. Here we determined that of the 37 OSNs found in *D. suzukii*, 32 out of 37 OSNs are functionally conserved between the two species (Figure 1A). We identified only five OSNs that showed robust functional deviation from the *D. melanogaster* model, including the ab2B-like, ab3A-like, ab9B-like, ab10A-like, and ai3A-like sensory neurons (Figure 1A). Data on the palps of *D. suzukii* were more difficult to confirm, for example, whether each sensillum contained only two OSNs. This is also owing to the reduced literature and molecular support available from other papers addressing the palps of most *Drosophila* species. However, we did not find enough evidence to determine that any additional OSNs were functional in the *D. suzukii* palp screens, though future studies will need to address this olfactory organ in more detail, especially pb1 sensilla. We also documented changes across ab5 and ab7 on the antennae of *D. suzukii*, though not as strong (i.e., weak), and we note that we found in total very few ab8 sensilla across *D. suzukii* trials. Two of the larger deviations in olfactory response for *D. suzukii* have been previously described, including ab2B and ab3A (Keesey et al., 2015). Interestingly, in the present study, we identified two distinctly responding ab3A OSN types, with an approximately 60:40 split ratio for OSN abundance, where the first (type i) was tuned toward isobutyl acetate (IBA) and the second (type ii) responded most strongly to beta-cyclocitral (βCC). In the last few years, increasing evidence has been provided to support the notion that the closest relative for *D. suzukii* is actually *Drosophila subpulchrella*, and not *Drosophila biarmipes* (Hickner et al., 2016; Ramasamy et al., 2016). Here we identify for the first time a second species that responds strongly to beta-cyclocitral, namely *D. subpulchrella* (Figure 4), which further supports an ecological the connection (Keesey et al., 2015; Piñero et al., 2019). Although the ecology of *D. subpulchrella* is understudied, based on the serrated ovipositor (Figure S7), it has been assumed that it also lays eggs in fresh or ripening fruit resources (Atallah et al., 2014; Ramasamy et al., 2016), just like the *D. suzukii* pest.

**Figure 2:**
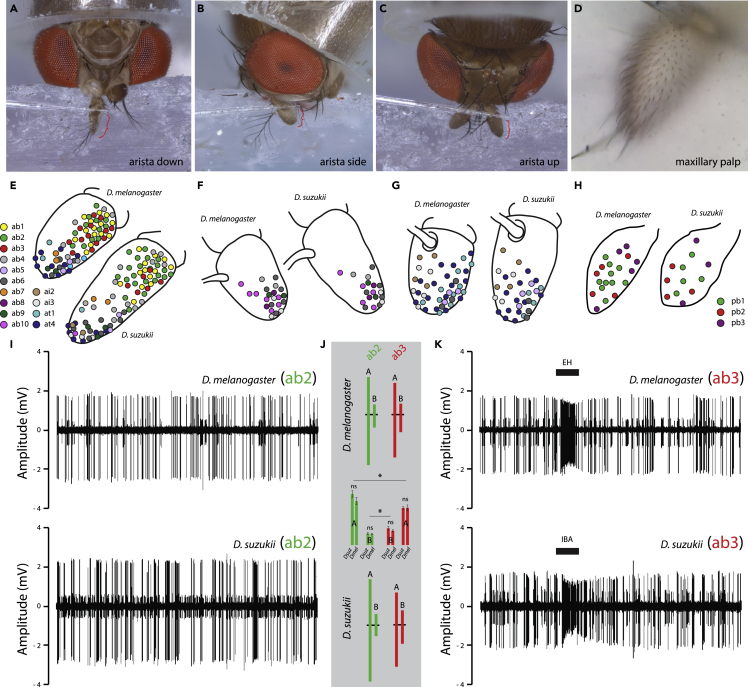
Olfactory sensory neuron mapping across the antennae of *D. suzukii* (A–D) The antennae were prepared in several different positions to optimize access to the various sensillar subtypes. (E) Schematic drawing of the third antennal segment from both species, where spatial distribution and sensillum abundance were identified during single sensillum recordings (SSR). Each color denotes a unique subtype. Shown are the sensillar mappings from the arista down configuration from both target species (*n* = 6–8 individuals). (F) Arista side configuration. (G) Arista up preparation. (H) Maxillary palp preparation. (I) Shown are example traces of the ab2 sensillum type, note maximum amplitudes well over 2 mV for each species. (J) Consistent ratio of amplitude differences between the A and B neurons for both ab2 and ab3 sensillum. Here there was a significant difference between amplitudes across receptor types, but not between species. Error bars denote SEM; normally distributed data were analyzed using two-tailed, paired *t*-tests (∗ denotes *p* < 0.05). (K) Example traces of ab3 sensillum type, where we note maximum amplitudes near 2 mV for each species. Here the relative size of the ab3B neuron amplitude is much larger when compared to ab2B, in relation to their respective A neurons for ab2 and ab3 sensillum types. In general, these stereotyped response dynamics appear conserved between species, and add an additional layer of confirmation to the chemical and morphological identification of sensillum type in novel *Drosophila* species. For scanning electron microscopy photos of *D. melanogaster* and *D. suzukii*, see related Figure S1. For details of electrophysiology data, see related Figure S2.

**Figure 3:**
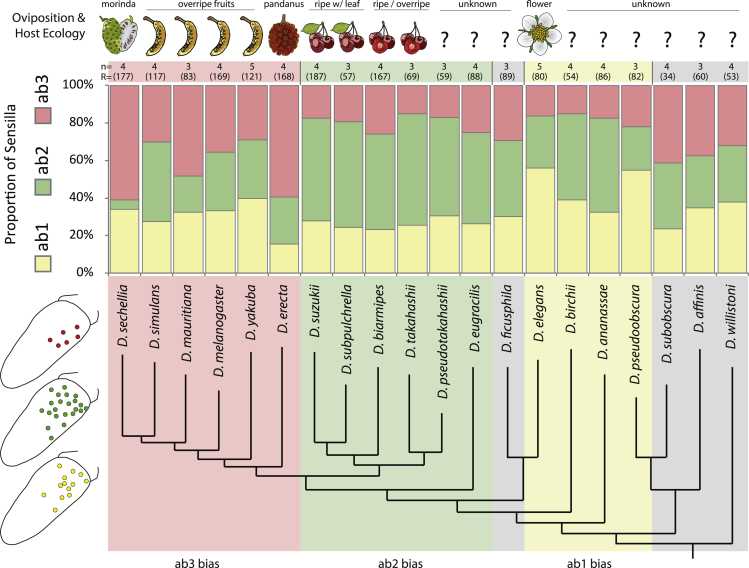
Large basiconic sensilla across the subgenus *Sophophora* The proportions of ab1 (yellow), ab2 (green), and ab3 (red) sensilla are shown for each of the 20 species examined, with total sample sizes of large basiconic sensillum contacts (R) listed in parentheses above each species, as well as the number of individuals that were pooled (*n*). In general, there is an increase of the ab3 sensillum type for the *melanogaster* clade (those in red on phylogeny), with extreme examples including *D. sechellia* and *D. erecta*, where we also note a drastic reduction in the ab2 sensillum type that is inversely related with the larger proportions of the ab3 type. In contrast, we notice an increase of the ab2 sensillum type across the spotted wing species, including the *suzukii* clade (those in green on phylogeny). Here, we see a relative reduction in ab3 that correlates with the increases for ab2 sensillum number. In addition (shown in yellow on phylogeny), there is a trend for increased ab1 representation in several species, whereas those species in gray denote a more even proportion for each of the three large basiconic types. For proportions of large basiconic sensillum types, see related Figure S11 and Table S1.

**Figure 4:**
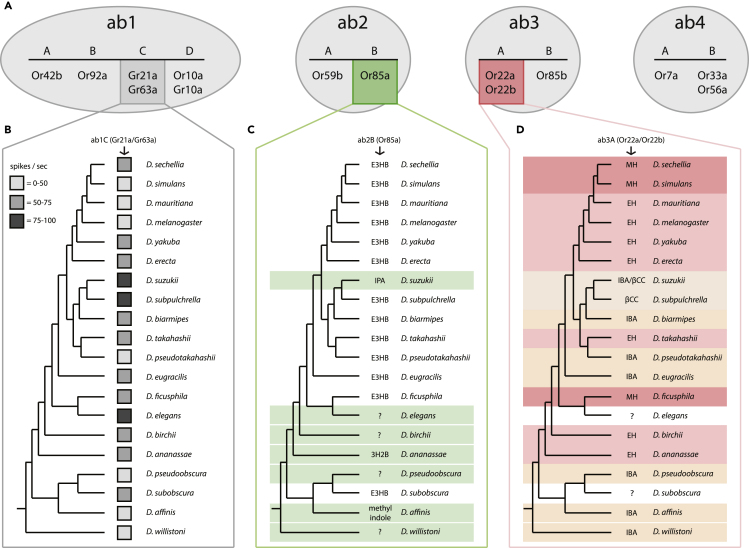
SSR and ligand spectra of four sensillum types across 20 species (A) Olfactory receptor neurons (OSNs) shown at the top without color were identical in their ligand responses across all 20 tested species (e.g. ab1A that houses Or42b in *D. melanogaster*). (B) Strong sensitivity variation noted for the ab1C neuron that contains the CO2 detecting GRs; however, strongest excitatory ligand was identical for all species. (C) Frequent ligand shift in the ab2B neuron (shown in green, which contains Or85a in *D. melanogaster*). Here the majority of species retained the exact same ligand spectra; however, 7 of the 20 species had an entirely novel ligand, which did not match with any of the other tested species, and we could not identify any odorant that activated this OSN in four different species. (D) We noted the most consistent changes in ab3A neurons between these 20 species (shown in red, which co-expresses Or22a and Or22b in *D. melanogaster*). Here we observed four or more separate ligand tunings, as defined by the strongest response at the 10–4 odorant concentration (diluted in hexane). For two species, we could not identify a strong ligand for this OSN, despite screening with over 80 synthetic compounds and thousands of natural compounds via GC-SSR high-throughput examination using a variety of plant, flower or fruit headspace materials (E3HB, ethyl-3-hydroxy butyrate; IPA, isopentyl acetate; 3H2B, 3-hydroxy-2-butanone; MH, methyl hexanoate; EH, ethyl hexanoate; IBA, isobutyl acetate; βCC, beta-cyclocitral) (*n* = 3–4 individuals). For olfactory responses of the ab2B and ab3Aneurons, see related Figures S12 and S13 and Tables S2 and S3.

Both of these ab3A-type OSNs in *D. suzukii* were tuned quite differently than those found within the corresponding sensillum of *D. melanogaster*, which is tuned instead toward ethyl hexanoate (EH). However, both species (and both types and type ii, in *D. suzukii*) share identical ligand spectra for the second OSN in the same ab3 sensillum, which responds characteristically toward 2-heptanol as the strongest excitatory ligand. We also found that although the ab2B OSN from *D. suzukii* deviates from *D. melanogaster*, the ab2A OSN appears identical. As such, we believe these two sensilla in *D. suzukii* (ab2-like and ab3-like) provide the strongest match for the comparative sensilla in *D. melanogaster*, despite the deviations in ligand spectra for select OSNs. This idea is further supported by the morphological structure of the sensillum (i.e., large basiconic), and by the large amplitudes that are characteristic of these sensillum types relative to the small basiconics. All these factors combine to strongly support the identity of these two sensillum types in *D. suzukii* adults, despite the variation in olfactory ligand spectra, as previously proposed (Keesey et al., 2015).

### Conservation of neuronal wiring despite ligand spectrum shifts

To continue to explore the neural pathways for the ab2- and ab3-like OSNs in *D. suzukii*, we next documented the circuitry from the antenna toward the antennal lobe (AL) using the single-sensillum backfill neuronal staining technique (Hansson et al., 1992). By comparing the labeled glomeruli from *D. suzukii* with the known AL atlas of *D. melanogaster* (Figures 1B–1E) (Grabe et al., 2016), we could provide additional support for the ab2 and ab3-like sensillum type identification, as these two fly species shared identical wiring for the corresponding OSNs, despite the observed deviation in ligand spectra for *D. suzukii* adults.

### Spatial mapping of sensillum subtypes

We next mapped the position of all sensillum subtypes on the *D. suzukii* and *D. melanogaster* antenna and palps using several individuals to create an aggregate diagram. The heads of both *D. suzukii* and *D. melanogaster* were positioned in four ways to completely map the spatial pattern for sensillum abundance, including antennal arista down, arista side, arista up as well as across the maxillary palp (Figures 2A–2D). Here, as we had established ligand spectra for all OSNs of both species, we used SSR data to document the position of each sensillum type on the antenna (*n* = 6–8 individuals), which provided spatial information as well as the relative abundance (where each dot represents the approximate location of each recorded sensillum), and these data matched previous studies of *D. melanogaster* antenna (Linz et al., 2013; Stensmyr et al., 2003). The positioning of each specific sensillum type was nearly identical in both species and arranged in concentric circles or zones; however, we did observe large variations in the abundance of sensillum types between species (Figures 2E–2H). For example, within the large basiconics, whereas the ab1 sensillum counts were nearly identical, we found that *D. suzukii* had almost twice as many ab2 as *D. melanogaster* (22 and 12, respectively). In contrast, we found that *D. melanogaster* had more than twice as many ab3 sensilla compared with *D. suzukii* (13 and 6, respectively). It was possible to identify large basiconics owing to physical metrics, like width, tip shape, and length (Figure S1). In addition, we could also positively identify large basiconics based on the amplitude of SSR spike responses (Figures 2I–2K). Here the ab2 and ab3 sensilla are also uniquely identifiable based on the ratio of the A neuron to B neuron spike sizes, where ab2 has an exaggerated disparity between the two neurons, whereas the ab3 neurons are much closer in spike size (Figures 2I–2K). Moreover, the ab1 sensillum is straightforward to identify owing to its unique housing of four OSNs, as well as the characteristic response to CO_2_ (Kwon et al., 2007). Whereas our sensillum counts are not the absolute total number of large basiconics, this was provided previously for these two species (Keesey et al., 2019a), and we are confident that the preparations and the zones of interest we counted from were the same during the comparison of the two species (Lin and Potter, 2015). As such, these counts represent strong relative values for species-specific comparisons of sensillar abundance. In addition, it is important to note that the flies do differ in absolute size, as does their antennal surface area (Keesey et al., 2019a, 2020). However, we still show a consistent difference that is not explained by the larger size of *D. suzukii* relative to *D. melanogaster* adults; therefore, additional evolutionary factors are in play concerning sensillum abundance.

### Proportional analyses of sensilla across *Sophophora*

As we were interested in the evolution of olfaction in *D. suzukii*, we next sought to examine 18 additional species within the *Sophophora* subgenus in order to generate an evolutionary framework of sensillar variation (Figure 3). Here we screened each new species for ab1-, ab2-, and ab3-like sensilla using a smaller panel of odorants from the original 80. The total sample sizes across individuals (*n*) and across large basiconic sensillum recordings (*R*) is listed above each species (Figure 3), and we present the data for different sensillum types as proportions of these total SSR basiconic contacts. As has been shown previously (Linz et al., 2013), we confirmed a large number of the ab3 sensillum for most of the *melanogaster* clade (Figure 3; ab3 increase; shown in red), including the confirmation of the most abundant ab3 species, *D. sechellia* and *Drosophila erecta*. Intriguingly, we also documented that each of the spotted wing species within the *suzukii* clade all displayed, in contrast to the *melanogaster* clade, a reduction in ab3 and a corresponding increase of the ab2-like sensillum (Figure 3; ab2 increase; shown in green). We believe the ab3 increase of the *melanogaster* clade might be linked to ecologically associated detection of strong fermentation components, whereas the ab2 sensillum is perhaps more tightly associated with fresh fruit esters or the fruit ripening process, but more work is needed to examine this in behavioral experiments. Our screen of the *Sophophora* subgenus also provided evidence for an ab1 increase in several species (Figure 3; shown in yellow). We do not have enough ecological information about many of these species, but we can speculate that perhaps there exists a common association with higher altitudes and altered CO_2_ concentrations in these alpine habitats. This, in turn, may help explain the ab1 abundance for some species (e.g., *Drosophila birchii*) (Griffiths et al., 2005), or that floral-, foliage-, and yeast-derived host signals might play a role in this association with CO_2_ sensitivity (Chandler et al., 2012; Cooper, 1960; Lachance et al., 1995). Lastly, four species had a roughly even proportion of basiconic sensillum types, which are shown in gray. Again, we are greatly limited owing to the unknown ecology of most species; thus, it is currently unclear what the evolutionary or ecological rationale could be for these differences in relative sensillum abundance.

### Functional ligand spectra for sensillum types across *Sophophora*

As we had established that some of the main olfactory differences between *D. suzukii* and *D. melanogaster* were related to large basiconics, we sought to test this hypothesis (i.e., that large basiconics play a consistent role in olfactory evolution) by looking at olfactory ligand variation across our 20 species within the *Sophophora* subgenus (Figure 4). Here we screened each species with a diverse panel of 80 odorants, as well as high-throughput testing that utilized fruit, foliage, and floral headspace extracts via gas chromatography-mass spectrometry combined with single-sensillum recordings (GC-SSR). Of the ten OSN types we examined from each of the 20 species, only three showed any dynamic variation in ligand spectra or ligand sensitivities (i.e., ab1C, ab2B, ab3A; Figure 4). We noted that the ab1C neuron (which co-expresses Gr21a and Gr63a in *D. melanogaster*) is narrowly tuned to CO2, although with varied sensitivities among the species tested (Keesey et al., 2015; Krause Pham and Ray, 2015; Pan et al., 2017; Robertson and Kent, 2009). Other neurons within this sensillum type, such as ab1A (which expresses Or42b in *D. melanogaster*), were functionally identical in each new species that we tested, with all species responding most strongly to ethyl acetate.

In general, ab3A (which co-expresses both Or22a and Or22b in *D. melanogaster*) was the most commonly changed OSN within the 20 fly species studied (Figure 4), and the response usually fell into one of four main categories of best odorant profile (i.e., methyl hexanoate (MH), ethyl hexanoate (EH), isobutyl acetate (IBA) or beta-cyclocitral (βCC)). The different ligands for ab3A often seemed to be conserved between closely related species. Further work is needed to confirm and reconstruct the neural circuit for these two sensillum types in *D. suzukii*, as while they map to the same glomerulus (DM2-like), it was unclear from our neuronal backfills whether types i and ii map to different regions within this same glomerulus. We note that several functional types of this ab3A OSN have been recently reported within *D. melanogaster* populations (Mansourian et al., 2018; Shaw et al., 2019). One variant expression is a chimeric gene, Or22ab, which arises from the fusion of both the Or22a and the Or22b gene products into a single receptor protein (Mansourian et al., 2018; Shaw et al., 2019). Moreover, we note that the ligand spectrum reported for this Or22ab variant is functionally similar to our current data in cases where we observe that IBA is the strongest excitatory ligand for many of these *Sophophora* species, including *D. suzukii*. Thus, it is possible that this variant receptor type also occurs within non-*melanogaster* members of this genus. Another consideration is that Or22c, which is usually expressed only in larvae, detects 2-acetylpyridine, which bears aromatic, chemical similarities to βCC (Mathew et al., 2013); therefore, another chimeric form, such as Or22ac, may also be possible in nature (Figures S2B–S2E). Overall, the olfactory changes for ab3A were more of a gradient or moderate shift between species, such as the transition from EH to MH as the strongest excitatory ligand (e.g., *D. melanogaster* and *D. sechellia*). We also note two species for which we could not identify any strong ligand for ab3A, *Drosophila elegans* and *Drosophila subobscura*. Here, not much is known about the ecology, and despite efforts to perform gas chromatography-coupled single sensillum recordings (GC-SSR) with a variety of headspace collections from flowers, fruits, and tree sap (including in total several thousand compounds), we could not identify any ligand candidates for this OSN in these two species.

Intriguingly, we also observed several changes in the ab2B neuron across our 20 examined species. In this case, most species responded to exactly the same ligand (i.e., ethyl-3-hydroxybutyrate (E3HB)). However, seven *Sophophora* members had an acute change in ligand spectra, where none of these seven species shared any overlap for their novel ligand, making each of these changes entirely species-specific. For example, as was published previously (Keesey et al., 2015), we again demonstrated that *D. suzukii* ab2B has changed ligand spectrum toward the detection of isopentyl (isoamyl) acetate (Figures 1 and 4), which is an odor associated more with ripe as opposed to overripe fruit. For two of the other six species with changes in this ab2B OSN, we could identify a strong ligand (Figure 4), including *Drosophila ananassae* (3-hydroxy-2-butanone; also known as acetoin) and *Drosophila affinis* (methyl indole and α-pinene); however, again, very little is known about the ecology of these two drosophilids. Despite the high throughput GC-SSR screening of the remaining four species, we did not identify any strong ligands. We believe that screening with host-related odors (once more ecological information is known) will most likely lead to the identification of ligands for all of these *Drosophila* species with functional changes in ab2B, but it is also possible that some of these olfactory receptors are non-functional pseudogenes owing to natural mutation in the amino acid sequence. Thus, at this stage, we cannot definitively say whether the variation in ab2B is (a) owing to a drastic ligand shift in the receptor (i.e., protein sequence variation), (b) owing to the replacement with a new or duplicated olfactory receptor (Prieto-Godino et al., 2020), or lastly, (c) a result of non-functional pseudogenes. However, at least in *D. suzukii* we have shown that the ab2B neuron, despite an acute shift in ligand spectrum, remains fully functional. Moreover, this OSN still maps via the same neural circuit to the same location within the AL (Figures 1B–1E), and thus might carry a similar behavioral relevance for *D. suzukii* as that of the *D. melanogaster* model.

### Receptor sequence alignments across the *Sophophora* subgenus

**Figure 5:**
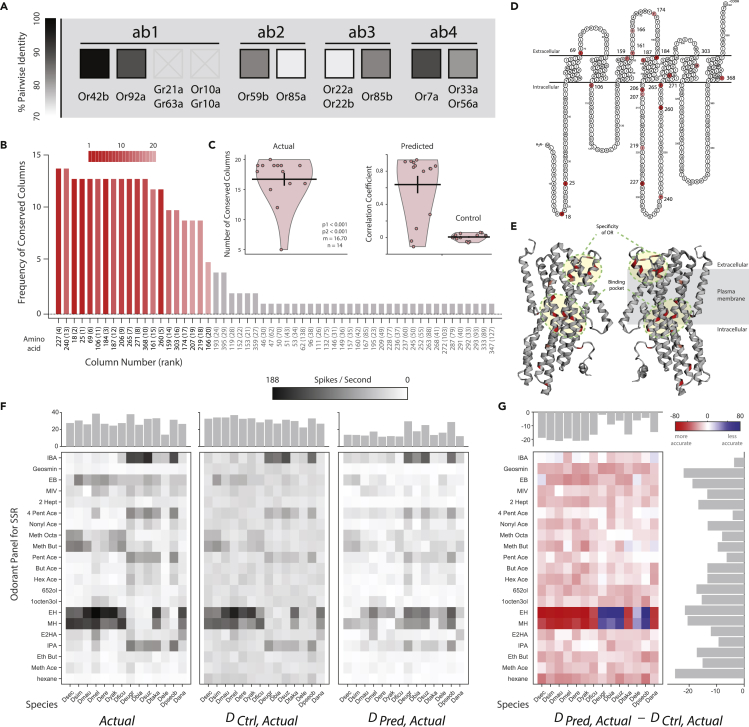
Protein sequence alignments of receptor orthologues (A) Heatmap (greyscale) between the sequences representing the percent pairwise identity between species. Darker colors illustrate higher similarity, and lighter colors denote receptor proteins with high variability. An X denotes receptors with insufficient sequence data available for comparison. (B) Identification of amino acid positions where changes were most consistent in their effect on receptor function for OR22a across 14 species. Each position was ranked using the full data set of 14 species (numbers in brackets, heatmap). As a test of robustness of this analysis, all sequence positions in the protein are sorted according to the frequency with which they appear among the top 20 positions re-calculated from each of the 14 reduced data sets (formed by excluding one species at a time from the full dataset). Note that the 20 most frequently seen positions in the recalculations match the original top 20 positions. (C) A second test of robustness of our analysis: each point shows the number of the original top 20 amino acid positions calculated from the full dataset that were retained in each of the recalculations from a reduced dataset. Note that a large fraction (∼17 out of 20, on average) of the original positions are retained in each recalculation. (D) The top 20 amino acid positions across the 2D structure of the *D. melanogaster* Or22a protein. (E) Predicted protein folding for Or22a sequence, with top 20 positions highlighted in red. Side view (through plasma membrane) of Or22a protein structure. (F and G) Heatmaps showing the relative prediction error (measured as D_Pred, Actual_ − D_Ctrl,__Actual_) for each species-odorant pair for the sets of 21 odorants and 14 *Drosophila* species. Note the high frequency of negative values (shown in red) and very low frequency of positive values (in blue), indicating that the modeled predictions were closer to the actual value than the control in most cases. Top inset: column averages depicting the predictive strength for the different species’ Or22a receptors; right inset: row averages depicting the predictive strength for each odor ligand. For protein sequence alignments, see related Figures S3, S4, S5, S6, and S10. For Trees based on sequence similarity, see related Figures S7 and S8. For the protein tertiary structure, see related Figure S9. For OR and GR accession numbers, see related Table S4.

As we had established both functional variation and conservation across OSNs within the *Sophophora* subgenus, we next sought to examine the structural basis of these evolutionary changes by analyzing the olfactory receptor (OR) protein sequences. Our assumption was that when functional SSR data did not vary between species, we would observe a lower number of amino acid changes in OR sequence orthologues. Thus, we first identified as many sequences from our 10 OSNs (e.g., those olfactory receptors housed (at least in *D. melanogaster*) in ab1, ab2, ab3, and ab4 sensilla) from as many of our 20 species as were publicly available in databases such as Flybase and GenBank. The alignment of these orthologues provided a wealth of information in conjunction with our SSR functional data screen. Here we observed that the response profile of ORs that were identical between our 20 species during SSR testing were indeed nearly identical at the amino acid sequence level (Figure 5A; e.g., Or42b). Although it has been suggested that the geosmin-detecting receptor, Or56a, is the most widely conserved receptor across the genus *Drosophila* (Stensmyr et al., 2012), here we observed that other ORs are equally or even more functionally conserved, such as Or42b, which is housed in the ab1A OSN and responds to ethyl acetate (an attractive odorant). We found very few amino acid changes in most receptors, which echoed our lack of functional shifts in ligand sensitivity or selectivity in our SSR datasets (Figure S2F; shown is Or42b; note high pairwise identity between species, in black). However, other receptors, such as those found in ab3A (i.e., Or22a/Or22b in *D. melanogaster*), which were highly variable between species in our SSR data, were also demonstrated here to be highly variable at the amino acid sequence level (Figure S2G; shown is Or22a; note low pairwise identity between species, in grey/white). The same trend was true for ab2B (which houses Or85a in *D. melanogaster*), where sequence variance mirrored the diversity in SSR response profiles between our species (Figures 5A and S3). Further phylogenetic analysis showed, in general, that olfactory ligand variation positively correlates with amino acid variation in the corresponding ORs (Figures S7 and S8).

The species-specific responses of these two sensillum types (ab3A and ab2B) also show up in a principal component analysis (PCA), with *D. suzukii* closely clustering with *Drosophila eugracilis*, *Drosophila pseudoobscura*, and *D. biarmipes* in their ab3A responses (Figure S9D). However, *D. suzukii* showed overall much less similarity regarding both SSR in ab2B-like sensillum and sequence data as compared with the other species (red dots; Figures S9F and S9G), indicating that Or85a (ab2B) may have been replaced by another receptor protein in this species (Prieto-Godino et al., 2020). We further observed that the sequence similarities tended to be relatively low within the transmembrane regions 1 and 3 (Figures S9H and S9I), suggesting that these regions of the protein might be associated with species-specific differences across the *Drosophila* genus, as was previously suggested from analyses of the *melanogaster* clade (Auer et al., 2020).

### Identification of response-determining positions in OR22a

Using an empirical approach described in (Chepurwar et al., 2019), we identified the top 20 amino acid positions that predict the odorant responses of ORs. These amino acid positions were determined using the response data from the OR22a receptors across 14 *Drosophila* species for which genomic sequence data were available (Figures 5 and S5A). To quantify the robustness of these top 20 positions, we recalculated the top positions by removing the data of one species at a time from the dataset (i.e., calculating top positions using a reduced dataset of only 13 species, where this recalculation was done 14 times by removing a different species each time). We then looked at two indicators of robustness: (1) which sequence positions appear most frequently among the top 20 positions recalculated from each of the 14 reduced datasets, and (2) how frequently the original top 20 positions calculated from the full dataset appear in each of the recalculations from the reduced datasets. For the first indicator, we found that the 20 most frequently occurring sequence positions in the recalculations from the reduced datasets were identical to the original 20 amino acid positions calculated from the full dataset (Figure 5B). For the second indicator, we found that about 17 of the original top 20 positions predicted from the full data set were retained, on average, in each recalculation from a reduced dataset (Figure 5C). Overall, these results suggest that the identified top 20 amino acid positions are robust to perturbations in the training data set, and therefore could serve as reliable predictors of OR22a responses across the genus.

### Locations of the top response-determining residues

To establish where these top 20 response-determining amino acid positions are located within the protein structure, we first visualized these positions in the secondary structure of OR22a of *D. melanogaster* (Figure 5E) using the Protter tool (Omasits et al., 2014). These residues were not limited to any one helix or loop, but were instead, found across several regions of the seven transmembrane proteins. Next, we checked the location of these amino acid residues within the 3D structure (Figures 5F and 5G), generated using the ORCO structure as reference ((Butterwick et al., 2018); SWISS-MODEL tool). Interestingly, we found that our predicted top 20 positions were concentrated in primarily two clusters across the 3-dimensional space, one at the entrance (which is suggested by (Butterwick et al., 2018) to be essential for preserving the specificity of the OR) and another cluster at the putative binding site (Auer et al., 2020). To quantify the concentration of top residues in the first region (entrance), we selected one residue in that region, R166, and checked all residues within 15 Å (angstrom) radius from it. Out of 41 residues present in the selected volume of the Or22a tertiary structure, 6 were among the top 20 response-determining positions, nearly three times the number expected by chance in a protein of length 397 (41 × 20/397 = ∼ 2.07; *p* = 0.011; hypergeometric test). One of these six positions (F159) has been previously shown to be important for determining olfactory responses by mutagenesis in *D. melanogaster* OR85b (orthologous position: Y150 (Nichols and Luetje, 2010)). In the second region (putative binding pocket), we selected one residue (F265) and checked all residues within a 15-Å distance. Here, out of 70 residues present in that surrounding volume, seven positions were among the top 20 response-determining amino acids, twice the number expected by chance (70 × 20/397 = ∼ 3.53; *p* = 0.044; hypergeometric test). One of these seven positions, A260, has also been shown to be important via mutagenesis in *Bombyx mori* OR1 (orthologous position: D299 (Nakagawa et al., 2012)). Overall, 13 of the top 20 amino acid positions were present within the two volumes containing only 111 of the 397 residues present in the protein (*p* < 0.0001; hypergeometric test), suggesting a significant role for these regions in determining the observed olfactory response variation between species.

This Or22a protein structure data support the changes that have been reported from several members of the *melanogaster* clade (Auer et al., 2020; Dekker et al., 2006). Here we find again similar amino acid positions that were previously identified, and which were predicted to be the putative binding pocket of the Or22a receptor (Figures 5E–5G, in red). However, it is not clear how these protein sequence alterations would affect protein folding and thus tertiary structure, therefore additional study is needed to address these hypotheses, especially as it relates to ligand selectivity and binding pocket function (Butterwick et al., 2018; Leary et al., 2012; Yang et al., 2017). As the cryo-EM structure of ORCO has now been elucidated (Butterwick et al., 2018), we anticipate a highly researched olfactory receptor such as Or22a would be the next viable candidate for crystallography and cryo-EM studies, and thus would allow for an expanded testing of the before-mentioned hypotheses about functional changes to binding pocket structure. Here we have used ORCO as the template in this analysis as at the time it was the only OR with known structure. More reliable homology modeling and stronger inferences will become possible using the same approach as structures of more similar ORs become available. A recent study has emerged utilizing advanced modeling techniques to examine ligand affinity and binding in three-dimensional protein structures from an OR of the jumping bristletail (*Machilis hrabei*), and their findings complement and enhance our findings and interpretations of potential chemosensory evolution (del Mármol et al., 2021). In the present study, we also addressed the protein sequences for the gustatory receptors that detect carbon dioxide (CO2; Gr21a/Gr63a; ab1C; Figures 4B and S10). However, as very few of the target species had genomic data, it remains unclear how these minor changes in sequences account for shifts in ligand sensitivity, where, again, all species detect CO2 but to a variable degree (Figures 4B and S10).

### OR22a selection analysis

To determine the selection regimen governing the evolution of the OR22a genes, we performed four different tests. BUSTED was used to test for selection across the complete OR tree as well as by selecting the OR22a and OR85a foreground branches with synonymous rate variation. BUSTED found no evidence (LRT, *p* value = 0.05) of gene-wide episodic diversifying selection in any of the branches of the OR22a and OR85a phylogeny, indicating that there is no evidence that any sites have experienced diversifying selection. To test if positive, lineage-specific selection has occurred on only a proportion of branches, signatures of diversifying selection were inferred using aBSREL. A total of 26 branches were formally tested for diversifying selection. aBSREL found no evidence of episodic diversifying selection in the OR phylogeny.

We performed the RELAX test to search for a putative signal of relaxation among the OR22a CDS, using as reference all branches of the OR phylogeny. RELAX Test for selection relaxation (*K* = 0.32) was significant (*p* = 0.000, LR = 162.68). Next, we used MEME (Mixed Effects Model of Evolution) to identify sites that have experienced episodic positive selection. Using a *p* value threshold of 0.05, MEME found evidence of episodic positive/diversifying selection at eight sites of the OR22a sequences. Positions 18, 166, 184, 207, and 227 overlap with sites that predict OR22a odorant responses using the empirical approach described in (Chepurwar et al., 2019). In addition, sites 32, 57, and 209 were only found in our analysis (Figure S5A).

### Response prediction using identified positions

Is it possible to predict the odorant responses of a novel receptor protein, if we already know the response of orthologous receptors in other closely related species? In our data set of responses for 14 orthologous OR22a receptors toward a set of odorants, we excluded the responses of one species at a time, and then predicted the responses of this excluded species using an adapted computational approach (Chepurwar et al., 2019). This earlier paper focused on predicting the olfactory response utilizing the responses of paralogous receptors within the same species, whereas in the present study, we base our predictions on orthologous receptors across closely related species. As previously described (Chepurwar et al., 2019), we used a shuffled control and a distance-based metric to assess the quality of our model predictions. We define $D_{\mathrm{Pr}ed,Actual}$ as the absolute difference between the predicted response and the actual response of an OR toward an odor, and $D_{Ctrl,Actual}$ as the absolute difference between the control prediction and the actual response. Thus, these two terms indicate the error in the actual and the control predictions. If our predicted response is better than the control prediction (e.g., closer to the actual observed response), the value of $D_{\mathrm{Pr}ed,Actual}-D_{Ctrl,Actual}$ should be negative (colored in red; Figure 5B). In each iteration, we excluded data from the receptor of a single species, then determined the top 20 residues using the remaining 13 receptors, and predicted the response of the excluded species using these top residues and the responses of the available 13 receptors; thus, predictions were made for the 14 receptors in 14 different iterations (Figure 5B).

The heatmaps in Figure 5 show the comparisons for all our modeled predictions, and reveal a very high abundance of negative values (shown in red), which indicates that our predicted response was better than the control. The average $D_{\mathrm{Pr}ed,Actual}$ of an OR (14.71) was smaller than the average $D_{Ctrl,Actual}$ (31.02) by 16.31 spikes, a predictive improvement of more than 53% over the control (Figures 5F and 5G). Thus, overall, our modeling predictions using the sequence similarity at the top 20 response-determining positions were significantly closer to the actual responses than control predictions. These predictive improvements were substantial considering the absolute values of odorant responses in OR22a from 14 *Drosophila* species (mean ± SD = 28.78 ± 33.48 spikes; *N* = 14 × 21 species-odorant combinations).

In summary, our modeling results show that the chemical responses of an olfactory receptor can be reasonably predicted using the available data from orthologous receptors (i.e., Or22a) across closely-related species when combined with functional characteristics (Figure 5). We also note that as more *Drosophila* species continue to be sequenced, the responses of orthologous receptors in new species can continue to be more accurately predicted using just their OR sequence or genomic data combined with our current olfactory response data set. This finding greatly extends the utility of the present study toward future novel species or novel olfactory receptor proteins within this genus.

## Discussion

Despite the fact that the first odorant receptors were described almost 30 years ago (in mammals (Buck and Axel, 1991); and insects (Vosshall et al., 1999)), and that the study of receptor–ligand interactions have been established using various expression systems (Hallem et al., 2004; Katada et al., 2003), it has remained elusive how chemical ligands interact with their corresponding receptors. Perhaps most importantly, the evolution of olfactory systems of related species occupying different ecological niches remained unexplored. Only recently, progress has been made regarding predictions of unique receptor–ligand interactions. For example, either for predicting a novel ligand based on chemical similarity using the ligand of interest and already established ligands of a similar receptor (Haddad et al., 2008; Schmuker et al., 2007), or for predicting a receptor function based on sequence similarities with another receptor using previously established ligands (Chepurwar et al., 2019). Here we analyze the olfactory response profiles of different olfactory receptors in 20 closely related *Drosophila* species. We first started by identifying all 37 OSN types in *D. melanogaster* and *D. suzukii* (Figure 1), and then highlighting those OSN types that deviate strongly in their olfactory function (e.g., ab2B, ab3A, ab9B, ab10A, and ai3A). We next characterized 10 OSN responses across another 18 species from the *Sophophora* subgenus (Figure 3), where we found that the ab2B neurons showed conserved responses to ethyl-3-hydroxybutyrate in the majority of the species; however, seven species exhibited completely non-overlapping response patterns in this OSN type. Here we propose that a series of olfactory receptor replacements is the most viable evolutionary explanation for this chemosensory variation across our 20 members of the *Sophophora* subgenus (Figure 4).

Contrary to the ab2B neurons, the ab3A neurons of the 20 total species exhibited only gradually changed chemical response profiles, suggesting that perhaps homologues of the same receptor (i.e., Or22a in *D. melanogaster*) are expressed in ab3A neurons of all species tested. By correlating these ligand response profiles with the species-specific Or22a receptor sequences (e.g., for those 14 species, where the sequences were available) we were able to localize the top 20 amino acid positions that determine the functional response profile of this receptor protein (Figure 5). Interestingly, 3D modeling of the receptor protein localized these 20 amino acids into two distinct spatial regions that had already been suggested to be involved in determining receptor specificity or ligand binding pocket (Auer et al., 2020; Butterwick et al., 2018) (Figures 5F and 5G). Although standard tests for positive selection across OR22a genes were negative, we found evidence for trends and/or shifts in the stringency of natural selection. Our finding that OR22a sequences bear putative signals of relaxation of purifying selection as well as episodic positive selection acting at specific functional sites of OR22a could be explained by the broader functional response profile of the OR22a receptor, permitting the evolution of new ecological adaptations (Andersson et al., 2015). In addition, based on the sequence locations for these 20 amino acid positions across the functional receptor of each species, we could begin to predict receptor function across novel *Drosophila* species with previously unmatched accuracy (Figure 5D).

An important observation from our dataset is that some OSN locations are far more likely to change than others, for example, we observe consistent alterations in ab2B and ab3A across our 20 species, whereas other OSNs remain functionally identical as well as continue to be highly conserved in their amino acid sequence (Figure 4). One explanation for these specific OSN changes could be related to yeast or host-specific microbial odorants. It has been shown previously for *Drosophila* species that yeast control attraction and oviposition more so than the host plant material (Becher et al., 2012); thus, it is possible that the repeated alterations in olfactory function of these OSNs, especially ab2B and ab3A, are related to yeast-specific and ecologically vital odors for each fly species (Koerte et al., 2020). Another possible explanation for the observation that elevated levels of amino acid sequence variation between species more consistently occurs for a particular OR could be related to the stability of tertiary structures, especially within transmembrane regions or putative binding pockets, or the stability during folding of the protein sequence itself (Chepurwar et al., 2019; Dalton et al., 2013). In a similar fashion, it is also possible that a stability explanation could account for why some amino acid regions within a protein sequence are also more likely to change. For example, it is unclear why amino acid positions 45, 67, or 93 of the Or22a sequence display lower levels of sequence conservation between six species within the melanogaster clade (Auer et al., 2020), a general region we also show is consistently variable across our 20 examined species (Figure 5). It could also be argued that relaxed constraints at the amino acid sequence level are not necessarily reflected at the level of tertiary structure in cases where the amino acid changes are conserved (i.e., basic amino acids are exchanged for another basic amino acid). Here, less conserved parts of the ORs might carry many changes in coding sequences that are neutral since they do not affect function. In cases with ligand shifts, instead, the observed amino acid changes could be in positions that allow variability in ligand binding, potentially changing the odorant perceived (i.e., bound) by a particular OR. Moreover, it is likely that several specific regions of the protein sequence and corresponding tertiary structure are excellent candidates to further examine how specific amino acid substitutions relate to ligand spectra shifts between species, or perhaps toward the stability of ligand binding affinities within the binding pocket (Auer et al., 2020; Dalton et al., 2013; Shykind et al., 2004).

Since its introduction into Europe in 2008, *D. suzukii* has been extensively studied, primarily as an agricultural pest, but also as an emerging model for speciation and evolutionary neuroethology (Crava et al., 2016; Dekker et al., 2006; Hickner et al., 2016; Karageorgi et al., 2017; Keesey et al., 2015, 2019a). Here we provide significant progress toward the functional characterization of all OSNs in both the antenna and the palps of this adult fly. Surprisingly, 32 out of 37 OSNs that we compare between *D. suzukii* and the classical model, *D. melanogaster*, show conserved odorant binding affinities. Thus only five OSNs deviate strongly in their olfactory function, including ab2B, ab3A, ab9B, ab10A, and ai3A, where several of these receptors in *D. suzukii* were predicted to be functionally divergent, according to the analyses of genomic data alone (Crava et al., 2016; Hickner et al., 2016; Ramasamy et al., 2016). For this study, we primarily focus on the two large basiconic sensillum types (e.g., ab2 and ab3), and further document that, although their odorant tuning differs, *D. suzukii* appears to maintain the same neural connectivity to the primary olfactory processing centers within the AL (Figures 1B–1E). However, it was shown recently in *D. simulans* that even when peripheral odorant spectra are conserved, changes in neural circuitry can occur in the higher brain regions, and can lead to behavioral valence shifts (Seeholzer et al., 2018). Thus, additional work should be conducted to assess neural connections in *D. suzukii* for variations that may not be apparent at the periphery or within the input side of the AL.

Another interesting phenomenon that we observe is that there are seemingly two different functional types of the ab3A-like neuron in this pest insect, one that is tuned toward IBA, and the other toward βCC, where both functional types are found in the same animal. This suggests that sensillum types across the antenna are non-uniform, and that OR gene duplication events and sequence divergence are potentially important, perhaps playing a wider role in the evolution of species-specific use of olfactory cues (e.g., Or22a, which has six functional duplications in *D. ananassae*). Here we propose that variation of expression may exist between gene duplications, and could vary between individuals within the same population. Alternatively, duplication events may allow new copies to have relaxed constrains on the amino acid sequence as long as the original OR retains its function. However, we again note that the ab3B OSN remains identical between these two types of ab3 in *D. suzukii* as well as identical to that recorded from *D. melanogaster* adults (i.e., detecting 2-heptanol). Thus, both ab3B (Or85b) and ab2A (Or59b) can act as anchors for identifying ab2-like and ab3-like sensilla within all 20 examined *Drosophila*, as these OSNs are highly conserved and seemingly identical throughout our *Sophophora* phylogeny despite the paired OSN in the same sensillum often shifting ligand affinity (Figures 4 and 5).

Here we posit that receptor copies may be differentially expressed (in location and relative abundance) across the antenna, potentially promoting disparities in ecological preferences. This may also support observations of behavioral and neuronal differences between individuals within a single species (Honegger et al., 2019; Linneweber et al., 2020). A similar duplication was shown previously to occur with the Ir75 complex (Prieto-Godino et al., 2017), in this case within *D. melanogaster*, where these co-expressed OSNs map to the same glomerulus, but to differing neural regions or spatial locations. Whereas our two ab3A OSN types appear to map to the same glomerulus, it is unclear whether they map to different regions within this single glomerulus. Future studies are needed to test the hypothesis that a single glomerulus can split into two or more during the evolution of new odorant function at the periphery or vice versa (Chai et al., 2019; Prieto-Godino et al., 2017). Again, we also propose that a developmental exchange between larval and adult copies of Or22a/Or22b and Or22c could also account for *D. suzukii* adults detecting βCC, as this odorant matches closely with the best-known ligand for Or22c (a receptor usually only expressed in larval stages for *D. melanogaster* and that detects 2-acetylpyridine) (Mathew et al., 2013). In *D. melanogaster*, many ORs are adult- or larval-specific, and are only functionally expressed during those developmental stages. Here we posit that these stage-specific OR expression patterns may vary between species. To our knowledge, genetic comparisons of olfaction across species are generally done from adult or larval tissues only, but we hypothesize that evolutionary gains and losses of receptors may be better explained through the inclusion of expression patterns from both life stages for more species. In general, it is possible that larval and adult stages within a species display different olfactory preferences to reduce competition between themselves (Dweck et al., 2018; Koerte et al., 2020). In addition, future examination of this stage-dependent phenomenon could potentially address the order or sequence of events that facilitate new olfactory and ecological niches (Keesey et al., 2020; Koerte et al., 2020; Prieto-Godino et al., 2020; Vogt et al., 2020).

Besides the similarity of odorant responses across the species of the *suzukii* clade, we also note for the first time an increase of the ab2-like sensillum within this genus (Figure 3), which is in contrast to the well-studied *melanogaster* clade that instead has a general increase in the ab3-like sensillum. As such, we continue to compile additional strong support for the notion that relative size and energy allocation toward a particular odorant is indicative of ecological relevance (Dekker et al., 2006, 2015; Keesey et al., 2019a; Linz et al., 2013). Here it appears that *D. suzukii* and other spotted-wing relatives have enhanced their fresh fruit detecting OSNs at the cost of those that detect fermentation by-products (Figure 3). Moreover, we also show an additional shift in ligand spectra for two fermentation-related neurons, ab2B and ab3A, which, in turn, now detect ripening fruit odors (Figures 1 and 4), thus seemingly pushing the *suzukii* clade further toward an ecological niche and host preference that is different from the *melanogaster* clade.

As more effort is devoted toward research into the relatives of *D. melanogaster*, an assumption has been made that this species group is an ecological model for the entire *Drosophila* genus. However, current data illustrate that the *melanogaster* clade is in fact the most divergent from the other members of the *Sophophora* subgenus, where most of the tested species, as well as the more basal species in the phylogeny, show an ab3A odorant tuning toward IBA and not EH or MH odorants (Figure 4). Here we also note that most basal species also have different ligands for the ab2B OSN (Figure 4). Thus, our data mirror the hypothesis that the adaptation of the *melanogaster* clade toward human commensalism and a cosmopolitan lifestyle, which is built around fallen, fermenting fruit, perhaps related to human agriculture, is purported to be a more recently derived phenotype for this subgroup (Mansourian et al., 2018). Similarly, it is likely that the ecological preference for fermented, as opposed to ripe fruit, has perhaps evolved multiple times across the *Sophophora* subgenus, given that we observe odorant tuning toward EH and MH several times within our screen of 20 species (Figure 4). Moreover, we note this potential fermenting fruit preference in *D. ananassae*, *D. birchii*, *Drosophila ficusphila*, and again for *Drosophila takahashii*, in addition to the six members of the *melanogaster* clade that we examine. At least for *D. takahashii*, a member of the *suzukii* clade, this fermentation preference was tested and confirmed in previous behavioral trials, where this species preferred to oviposit in fermented strawberries as opposed to ripe fruits (Karageorgi et al., 2017). As such, an additional study is required to continue to test our hypotheses about the ecological rationales for ligand spectra shifts in these other understudied *Drosophila* species. However, we feel the functional, olfactory evidence we provide in the present study supports the idea that ab3A (e.g., Or22a/Or22b in *D. melanogaster*) is strongly associated with host choice, either for feeding or perhaps indirectly for oviposition preferences as well.

In addition to the changes documented for the ab3A-like OSN, we also show a series of changes throughout our 20 *Sophophora* species for the ab2B-like OSN. In *D. melanogaster*, this OSN expresses Or85a, and whereas we find 13 of the 20 species retain the same ligand as *D. melanogaster* (i.e., E3HB), we also uncover seven species with widely varied ligand spectra for this OSN (Figure 4). Recently, it was predicted for *D. suzukii* that Or85a was lost during evolution, perhaps owing to pseudogenization (Hickner et al., 2016; Ramasamy et al., 2016). However, we clearly show that a fully functional OSN is present in ab2B (Figures 1 and 4). Thus, several options exist to explain this occurrence. Here we contend that the similarity in response to different ligands of OR22a between species contrasts with the results obtained with ab2B/Or85a, which responds to an entirely unique odorant in *D. suzukii*, and is narrowly tuned. Thus, several options exist to explain this occurrence.

First, it is possible that Or85a is a non-functional pseudogene that is not expressed in *D. suzukii* or that this OR is not transported to the cell membrane by the Orco chaperone. In this case, it would be likely that a different receptor is instead expressed in this OSN location as a replacement, and that this new receptor provides the unique SSR response profile that we describe (Prieto-Godino et al., 2020). In this scenario, the closest olfactory receptor match for the observed ligand spectrum in *D. suzukii* would be Or47a from *D. melanogaster*, which is co-expressed with Or33b within the ab5B sensillum subtype. Whether the co-expression of ORs within an OSN is correlated with receptor replacement has yet to be tested. Similarly, we suggest that other species may have also replaced Or85a within the ab2B OSN. For example, we predict that *D. affinis* may have replaced Or85a with Or43a, given the response to methyl indole. Moreover, in *D. ananassae,* Or85a may have been substituted with Or9a, given the ligand specificity toward 3-hydroxy-2-butanone.

Second, it is also possible that Or85a is only predicted to be a non-functional pseudogene from the genomic analyses of *D. suzukii* owing to a premature stop codon. In this case, a shortened sequence for Or85a in *D. suzukii* might retain functional expression, albeit with a completely unique ligand spectrum, and thus Or85a acts as a pseudo-pseudogene, which is a phenomenon that has been previously described in *D. melanogaster* (Prieto-Godino et al., 2016). However, this has only been documented for an ionotropic glutamate receptor (IR), and the present suggestion could be the first known case of an OR that acts as a pseudo-pseudogene. In either case, we demonstrate that a functional receptor exists at this OSN location in *D. suzukii*, and that the ligand spectrum deviates strongly from that found in *D. melanogaster*. Interestingly, we also document this same type of occurrence for ab2B in six other *Drosophila* species from our screen (Figure 4), where in each case, the ligand spectrum is entirely species-specific, and does not overlap with any other known species. This is unlike the slow changes or gradual shifts observed in ab3A/Or22a (Figure 4), where olfactory deviations in ligand spectra are often shared across several species. However, an additional possibility for ab3A is that a duplicate copy of Or22a, which contains several premature stop codons and therefore looks like a pseudogene, could be, in fact, a pseudo-pseudogene as well (Ramasamy et al., 2016). This new copy of Or22a could then be expressed in one of the two ab3A subpopulations of *D. suzukii*, and change or alter the response of the neuron. With all this in mind, whatever the cause for this change in the ab2B and ab3A OSNs, we show it occurs repeatedly, and that it is therefore likely to provide a new avenue for rapid olfactory evolution throughout the *Sophophora* subgenus (Figure 4). Recent research into *D. melanogaster* has also highlighted an additional large basiconic, currently named abx(3), that is indistinguishable from ab3 based on morphological and morphometric features, though its receptor identity and function have yet to be characterized (Gonzales et al., 2021).

Throughout the previous examinations of the olfactory system of several species within the genus *Drosophila*, several mechanisms have been proposed for the evolution of chemosensory receptors. It has been demonstrated that alterations in the neural circuitry of the P1 neurons (or neurons in higher brain centers) can dictate attraction or aversion for different species, despite the conserved peripheral detection of an odorant (Seeholzer et al., 2018). There have also been numerous publications describing the net gains or losses of chemosensory receptors that result in dramatic changes to host or habitat preferences, such as the variations shown for the *Scaptomyza* leaf-mining genus, which are still within the Drosophilidae family, but have, for example, lost Or22a entirely (Goldman-Huertas et al., 2015). In addition, there has also been a plethora of examples describing alterations in the relative abundance of chemosensory gene expression, either increases or decreases, where each change corresponds with ecological specialization in regards to either host or courtship preferences (Auer et al., 2020; Dekker et al., 2006; Keesey et al., 2019a; Linz et al., 2013). In these cases, the peripheral abundance also always accompanies a corresponding shift in glomerulus volume within the AL (Grabe et al., 2016; Linz et al., 2013). In the present study, we provide several new examples of the latter two of these mechanisms, both by illustrating a ligand spectrum shift at the periphery and providing robust evidence for sweeping changes in the relative abundance of OSN types. Intriguingly, we find that these two mechanisms often co-occur, that is, when ligands change their binding affinity, we also notice changes in relative sensillum abundance connected to those same OSN types. As such, it is difficult to determine what changes first in the timeline of olfactory evolution, ligand specificity, or receptor expression. Therefore, future studies of *Drosophila* should continue to compare closest relatives or entire clades of species in order to determine the potential chronology of evolution, which may or may not have a consistent temporal mechanism within this genus.

In this study, we highlight that understanding the evolution of olfaction requires not just the determination of which chemical odorant is detected by a given OSN, but also how the relative abundance of those detectors combines to generate a more holistic view of olfactory function within the *Drosophila* phylogeny. We identify several repeated examples of functional chemosensory evolution. First, we show that receptor expression can vary according to changes in relative sensillum number, for example, between the ab2- and ab3-like morphological subtypes. Second, we observe that a receptor can vary in its sensitivity toward the same odorant across our insect species (e.g., CO_2_). Third, we show that few amino acid sequence changes can lead to variations in ligand affinity that are often shared between close phylogenetic relatives (i.e., Or22a). Lastly, from our data, we identify drastic changes in odorant detection within the ab2B sensillum that, in *D. melanogaster*, contains Or85a. Here we propose that a series of olfactory receptor replacements is the most viable explanation for this chemosensory variation across our 20 members of the *Sophophora* subgenus. Importantly, we emphasize that all chemosensory changes are highly targeted, and repeatedly affect only the same subset of genomic and neural locations. These locations may commonly correlate with gene duplications events or sites of receptor co-expression (Auer et al., 2021), which may serve as potential mechanisms for ecological and evolutionary shifts in host preference. Thus, olfactory evolution across these 20 species appears to be highly repeatable in regards to where chemosensory changes consistently occur. Future studies should continue to examine all possible mechanisms to explain why some OR OSN locations are far more likely to shift than others within the *Sophophora* subgenus.

It is important to note that the persistent dearth of viable ecological and natural history information for the majority of the known *Drosophila* species makes the subsequent extrapolation toward evolutionary pressures, ecological mechanisms, or roles of niche partitioning difficult (Keesey et al., 2019c, 2020). This paucity of ecological and host information leaves several species without known ligands for either ab2B or ab3A, despite a chemically diverse and robust screening of these OSNs in the present study using known host materials from other members of this subgenus. As such, we continue to implore the expansion of scientific research to include a wider array of non-*melanogaster* species, especially those investigations that could provide ecological, host, and environmental, habitat, or behavioral rationales for morphological and chemosensory variation. We expect that as increased efforts are placed on additional *Drosophila* species, in future, we may identify and test additional hypotheses or explanations for the observed olfactory shifts within this incredibly diverse genus of flies. Through the utilization of the comparative method to study sensory biology (i.e., auditory, visual, olfactory, gustatory, and tactile cues) and behavioral ecology (i.e., feeding, oviposition, attraction, and aversion), we can continue to increase our understanding of the fundamental mechanisms by which evolution shapes the nervous system.

### Limitations of the study

We tested many aspects of functional olfaction, including relative abundance of sensillum types or estimates of olfactory receptor copy numbers within and between many different species. We also tested chemosensory sensitivity toward the same odorant or chemosensory variation in the strongest excitatory ligands for a particular OSN or location. However, we did not test any behavioral variables. Thus, it will be important in the future to address how (and if) biological variance in sensitivity or ligand type (e.g., changes in response to CO_2_ or EH/MH odorants) predicts valence (i.e., attraction versus aversion) and the strength of any induced activity at the organismal level. These behaviors will also be critical to better understand concentration dependent valence, where high levels can be attractive to some but not all species that functionally detect the same odorants. It is also worth noting that whereas we test several replicates across individuals within a species, population variance does occur, including genetic variability in OR type and function, even in extremely well-studied model species such as *D. melanogaster*. Future research will be required to confirm whether the species-specific differences we identify and describe are typical of all populations of the species we focused upon in this study.

## STAR★Methods

### Key resources table

**Table:** 

REAGENT or RESOURCE	SOURCE	IDENTIFIER
**Experimental models: Organisms/strains**		

*D. sechellia* (Dsech)	NDSSC	14021-0248.07
*D. simulans* (Dsim)	NDSSC	14021-0251.01
*D. mauritiana* (Dmaur)	EHIME-Fly	E-18901
*D. melanogaster* (Dmel)	Hansson Lab Strain	
*D. yakuba* (Dyak)	NDSSC	14021-0261.38
*D. erecta* (Dere)	NDSSC	14021-0224.01
*D. suzukii* (Dsuz)	NDSSC	14023-0311.01
*D. subpulchrella* (Dsubpul)	EHIME-Fly	E-15201
*D. biarmipes* (Dbia)	NDSSC	14023-0361.10
*D. takahashii* (Dtaka)	EHIME-Fly	E-12201
*D. pseudotakahashii* (Dpsetaka)	EHIME-Fly	E-24401
*D. eugracilis* (Deug)	EHIME-Fly	E-18101
*D. ficusphila* (Dficus)	EHIME-Fly	E-13301
*D. elegans* (Dele)	EHIME-Fly	E-13201
*D. birchii* (Dbir)	EHIME-Fly	E-24201
*D. ananassae* (Danan)	NDSSC	14024-0371.12
*D. pseudoobscura* (Dpseob)	NDSSC	14011-0121.00
*D. subobscura* (Dsubob)	NDSSC	14011-0131.04
*D. affinis* (Daff)	NDSSC	14012-0141.00
*D. willistoni* (Dwil)	NDSSC	14030-0811.24

### Resource availability

#### Lead contact

Lead Contact: Prof. Dr. Bill S. Hansson (Hansson@ice.mpg.de).

This study did not generate new unique reagents.

#### Materials availability

Further information and requests for resources and reagents should be directed to and will be fulfilled by the Lead Contact.

### Experimental model and subject details

Flies were obtained from The National Drosophila Species Stock Center, NDSSC, at Cornell University (Ithaca, USA), or from Ehime University (EHIME-Fly; Matsuyama, Japan). Stock numbers and reference specimens include the following genetic lines: *D. sechellia* (14021-0248.07), *D. simulans* (14021-0251.01), *D. mauritiana* (E-18901), *D. melanogaster* Canton-S (Hansson Lab Strain), *D. yakuba* (14021-0261.38), *D. erecta* (14021-0224.01), *D. suzukii* (14023-0311.01), *D. subpulchrella* (E-15201), *D. biarmipes* (14023-0361.10), *D. takahashii* (E-12201), *D. pseudotakahashii* (E-24401), *D. eugracilis* (E-18101), *D. ficusphila* (E-13301), *D. elegans* (E-13201), *D. birchii* (E-24201), *D. ananassae* (14024-0371.12), *D. pseudoobscura* (14011-0121.00), *D. subobscura* (14011-0131.04), *D. affinis* (14012-0141.00), *D. willistoni* (14030-0811.24). All fly stocks were maintained on standard diet (normal food) at 22°C with a 12 h light/dark cycle at 40% humidity (Keesey et al., 2019a). In addition, the following species had their diet supplemented with freshly crushed blueberries: *D. sechellia*, *D. suzukii*, *D. subpulchrella*, *D. biarmipes*, *D. takahashii*, *D. pseudotakahashii*, *D. ficusphila*, *D. elegans*, *D. pseudoobscura*, *D. subobscura* and *D. affinis*. Fly vials were maintained with consistent numbers of founding females (15–20) per container, in order to maintain consistent adult sizes via controlled population density. Phylogenetic information for all species was made available from previous publications as well as other literature (Crava et al., 2016; Ramasamy et al., 2016). Additional information about each organism is available through the Key resources table as well as the websites for ordering *Drosophila* species stocks.

### Method details

#### Chemical stimuli and single sensillum recordings

All synthetic odorants that were tested were acquired from commercial sources (Sigma, www.sigmaaldrich.com and Bedoukian, www.bedoukian.com) and were of the highest purity available. Stimuli preparation and delivery for electrophysiological experiments followed previously established procedures, and any headspace collection of plant, fruit or microbial volatile odors was carried out according to standard procedures (Keesey et al., 2015, 2017). For SSR experiments, 10μL of a dilution (10^−4^ in hexane) of an odor was loaded onto a filter paper disc that was placed inside a glass pipette. Electrophysiological contacts were made with tungsten electrodes (reference electrode into the eye, recording electrode into a single sensillum). Females were used from all species, and flies were between 2 and 7 days post-eclosion. An odor panel of 80–90 compounds was selected based on previous literature (de Bruyne et al., 2010; Ebrahim et al., 2015; Karageorgi et al., 2017; Keesey et al., 2015; Piñero et al., 2019), and was used to screen all OSNs across the antenna and palps of each examined species. In addition, when this odor screen failed to identify any strong ligands, we also utilized gas-chromatography single-sensillum recordings (GC-SSR). Here we employed odor collections from diverse floral, fruit, and microbial origins. In total, we estimate our GC-assisted screens included between 3000 and 5000 separate odorants, similar to previous studies (Dweck et al., 2016).

#### Neuronal staining (single-sensillum backfills)

Flies were prepared as usual for SSR inside a plastic pipette tip, and sensilla were identified first with characteristic odor screening using tungsten electrodes. The tungsten recording electrode was then removed and replaced with a pulled glass capillary which contained a filament, where the filament was pre-filled with neurobiotin via dipping the unsharpened end of the capillary into a 1–2% solution of the dye. Contact with the targeted sensillum was re-made on the SSR table with this filled glass electrode, which punctured, but did not pierce through both sides of the sensillum. A good contact was established when viable SSR spikes were observed, and when appropriate odor responses could be generated using this glass electrode (which replaced the tungsten wire). Light illumination was then removed, and this sensillum contact was maintained for 30–45 min, with periodic odor puffs to help push the dye towards the antennal lobe (AL). Both the A and B neurons were stimulated with their relevant odors every 5–10 min for the duration of the contact with glass-filled electrode and neurobiotin. Thus, axonal projections of OSNs from ab2 and ab3 sensilla were identified by SSR followed by this neurobiotin backfill. Sensillum types in *D. suzukii* were identified by SSR with the diagnostic odors IPA/methyl acetate for ab2 and 2-heptanol/IBA for ab3. Next, the recording electrode was replaced with a pulled glass capillary (with filament) that was filled with neurobiotin (Invitrogen, 2% m/v in 0.25 M KCl). Neurobiotin was allowed to diffuse for 45–90 min under periodic stimulation with the associated odors for each sensillum type. Brains were then dissected in PBS and fixed in 4% paraformaldehyde (PFA) for 30 min at room temperature (RT), rinsed 3 × 15 min in PBS with 0.3% Triton X-100 (PT). This was followed by incubation with mouse monoclonal NC82 antibody (1:30, CiteAb, A1Z7V1) and streptavidin conjugated with Alexa Fluor 555 (1:500, S32355, Invitrogen) in 4% normal goat serum (NGS,) in PT (48 h at 4C). Samples were washed 4 × 20 min in PT, incubated overnight with Alexa633-conjugated anti-mouse (1:250, A21052, Invitrogen) in NGS-PT, then rinsed 4 × 20 min in PT and mounted in VectaShield (Vector Laboratories) (Prieto-Godino et al., 2017). Images were acquired with a Zeiss 710 NLO confocal microscope using a 40x water immersion objective. The *D. suzukii* DM2, VM5d, DM4 and DM5 glomeruli identity was assigned based on similar 3D glomerular position and shape within the AL, as compared to those of the *D. melanogaster* antennal lobe atlas (Grabe et al., 2016).

#### Mapping and counts of olfactory sensillum types

Adult flies were prepared as has been described previously for single-sensillum recordings (Keesey et al., 2015; Lin and Potter, 2015). A single adult was immobilized in a plastic pipette tip, with only the head protruding. The fly was positioned in one of four ways, in reference to the arista (e.g. arista down, up, side 1 and side 2) (Figures 2A–2D). This positioning allowed for consistent orientation of the sensillar zones along the antenna of each species, and enabled consistent counting of sensillum types across individuals. We observe concentric rings or circular organization of the different sensillum types, especially the large basiconics. In this case, the ab3 is usually in the slight depression of the 3^rd^ antennal segment, followed by round concentric zones that increase in first ab1 and then ab2 sensillum types, and subsequently the state of small basiconics such as ab4. Schematics of each species were produced based on contacts with each sensillum type, where a sensillum was identified using physical metrics (i.e. size, tip shape, width) (Figure S1) as well as ligand identities of each OSN, and in addition, the electrical response dynamics such as amplitude and relative ratio of OSN firing rates or size (Figures 2I–2K). We found all 20 species had similar morphological characters for large basiconics, as well as electrical response dynamics; however, the density or abundance of each sensillum type varied greatly between species, but not between individuals. Previous estimates of abundance ^19^ utilized a sample size of 30–40 contacts, whereas here, we attempted to make between 40 and 100 contacts on average per species (Figure 3). Our estimates of sensillum proportions match very with previous examinations (Linz et al., 2013), which were universally restricted to the *melanogaster* clade, thus all species beyond this group are to our knowledge, newly described here concerning sensillar proportions.

#### Fly images (SSR heads, wings and ovipositor)

Dispatched flies were mounted and views of the ovipositor (180x) were acquired as focal stacks on an AXIO Zoom V.16 (ZEISS, Germany, Oberkochen) with a 0.5x PlanApo Z objective (ZEISS, Germany, Oberkochen). The resulting stacks were compiled to extended focus images in Helicon Focus 6 (Helicon Soft, Dominica) using the pyramid method. Here we provided images of the serrated ovipositors of close relatives to *D. suzukii* in order to highlight the physical deviations in egg-laying potential (Figure S1), which has been described previously (Atallah et al., 2014). We also documented the differences in male wing pigmentation (32x), as *D. suzukii* and *D. subpulchrella* can be difficult to distinguish (Figure S7). Moreover, *D. subpulchrella* were shown recently in the most up to date phylogenetic analyses of this *Drosophila* clade to be the closest relatives to *D. suzukii*, as opposed to *D. biarmipes* (Dekker et al., 2015; Hickner et al., 2016; Keesey et al., 2019a; Ramasamy et al., 2016). Images were also compiled of dissected heads (128x) to illustrate the mounting preparations for sensillum counts (Figures 2A–2D).

### Quantification and statistical analysis

All images and drawings are originals, and were prepared by the authors for this publication. Figures were prepared via a combination of Syntech AutoSpike32 (v3.7), R Studio (version 1.2.5033), Microsoft Excel, Adobe Illustrator CS5, EzMol (v1.22), and Geneious Prime (v10.2.3). Statistics were performed using GraphPad InStat version v3.10 and Past v3.25 at α = 0.05 (∗), α = 0.01 (∗∗), and α = 0.001 (∗∗∗) levels. For transmembrane region predictions, several resources were utilized (TMHMM Server v2.0; http://www.cbs.dtu.dk/services/TMHMM/; TMpred Server – EMBnet; https://embnet.vital-it.ch/software/TMPRED_form.html), and any nucleotides converted to amino acid sequences were performed using ExPASy (SIB Bioinformatics Resource Portal; https://www.expasy.org/). A multiple sequence alignment of protein was generated using MAFFT (v7.402) with default setting. The final trees were reconstructed using a maximum likelihood approach in FastTree (v2.1.8) with -gamma option to rescale the branch lengths. The tree was visualized and processed in Figtree (v1.4.2). Numbers next to the tree branches indicate the support values. The scale bar for branch length represents the number of substitutions per site.

#### Positive selection analysis

A codon-based alignment of the OR coding sequences was generated using MUSCLE (UPGMA Cluster method) implemented in MEGA X (Version 10.0.5). BUSTED (Branch-site Unrestricted Statistical Test for Episodic Diversification) provides a gene-wide test for positive selection by asking whether a gene has experienced positive selection at minimum one site on at least one branch (Murrell, B et al. "Gene-wide identification of episodic selection." Mol. Biol. Evol. 32, 1365–1371 (2015)). Signatures of diversifying selection were inferred using aBSREL (adaptive Branch-Site Random Effects Likelihood) an improved version of the commonly-used "branch-site" models, as implemented in Datamonkey (https://www.datamonkey.org). Significance was assessed using the Likelihood Ratio Test at a threshold of p = 0.05, after correcting for multiple testing.

RELAX was useful for identifying trends and/or shifts in the stringency of natural selection (and not to test for positive selection as such) on a given gene. A significant result of k > 1 indicates that selection strength has been intensified along the test branches, and a significant result of k < 1 indicates that selection strength has been relaxed along the test branches (Wertheim JO, Murrell B, Smith MD, Kosakovsky Pond SL, Scheffler K. RELAX: detecting relaxed selection in a phylogenetic framework. Mol Biol Evol. 2015 Mar;32(3):820–32. https://doi.org/10.1093/molbev/msu400). RELAX Test for selection relaxation (K = 0.32) was significant (p = 0.000, LR = 162.68). We used MEME (Mixed Effects Model of Evolution) to identify sites which have experienced episodic positive selection (Murrell B, Wertheim JO, Moola S, Weighill T, Scheffler K, Kosakovsky Pond SL. Detecting individual sites subject to episodic diversifying selection. PLoS Genet. 2012;8(7):e1002764. https://doi.org/10.1371/journal.pgen.1002764). MEME was run in Datamonkey (https://www.datamonkey.org) using standard parameters and a p value threshold of 0.05.

## Data Availability

Data All data supporting the findings of this study, including methodology examples, raw images and z-stack scans, molecular sequences, accession numbers, statistical assessments, phylogenetics as well as species information are all available within the online version of this paper or through Edmond, the Open Access Data Repository of the Max Planck Society: https://edmond.mpdl.mpg.de/imeji/collection/ahf5vh_AmtVBgWnI. Code This paper does not report original code. Any additional information required to reanalyze the data reported in this paper is available from the lead contact upon request.

## Supplemental information

Document S1. Figures S1–S13

Table S1. Sensillum counts and ratios across each species, related to Figure 3

Table S2. SSR odor panel screen for ab3A and Or22a, related to Figure 4

Table S3. SSR odor panel screen for ab2B and Or85a, related to Figure 4

Table S4. OR and GR accession numbers for sequence data, related to STAR methods, Figure 5, and Figures S3–S6 and S10
